# Transcriptional repression of IKKβ by p53 in arsenite-induced GADD45α accumulation and apoptosis

**DOI:** 10.1038/s41388-018-0478-7

**Published:** 2018-09-03

**Authors:** Yongliang Hu, Rui Jin, Ming Gao, Huan Xu, Shuxian Zou, Xiaoguang Li, Chen Xing, Qiyu Wang, Hongli Wang, Jiannan Feng, Meiru Hu, Lun Song

**Affiliations:** 1 Department of Neuroimmunology, Beijing Institute of Brain Sciences, 27 Taiping Road, Beijing, 100850 China; 2 Department of Tumor Biology, Beijing Institute of Biotechnology, 27 Taiping Road, Beijing, 100850 China; 3 State Key Laboratory of Environmental Chemistry and Ecotoxicology, Research Center for Eco-Environmental Sciences, Chinese Academy of Sciences, 18 Shuangqing Road, Beijing, 100085 P. R. China; 4 Guangxi Medical University, 22 Shuangyong Road, Nanning, 530021 China; 5 Anhui Medical University, 81 Meishan Road, Hefei, 230032 China; 6 Present Address: Department of Dermatology, The 309 Hospital of PLA, 17 Heishanhu Street, Beijing, 100091 P. R. China; 7 Present Address: Department of Breast Surgery, Fudan University Shanghai Cancer Center, Fudan University, 270 Dong’an Road, Shanghai, 200032 P. R. China

**Keywords:** Stress signalling, Apoptosis

## Abstract

Our previous studies revealed that GADD45α is a liable protein, which undergoes MDM2-dependent constitutive ubiquitination and degradation in resting HepG2 hepatoma cells. Arsenite exposure induces ribosomal stress responses mediated by the ribosomal protein S7, which can block MDM2 activity and result in GADD45α accumulation and cell apoptosis. In the present study, we found that one of the catalytic subunits of IκB kinase (IKK), IKKβ, exerted a novel IKKα- and NF-κB-independent function in stabilizing MDM2 and therefore contributed to ubiquitination-dependent degradation of GADD45α in resting HepG2 cells. Arsenite stimulation induced transactivation of p53, which formed a complex with its downstream target, Ets-1, and then synergistically repressed *IKKβ* transcription, reduced MDM2 stability, and ultimately removed the inhibitory effect of MDM2 on GADD45α induction. In addition, DAPK1 functioned as an upstream protein kinase triggering p53/Ets-1-dependent IKKβ and MDM2 reduction and GADD45α accumulation, thus promoting apoptosis in HepG2 cells. Subsequent studies further revealed that the activation of the DAPK1/p53/Ets-1/IKKβ/MDM2/GADD45α cascade was a common signaling event in mediating apoptosis of diverse cancer cells induced by arsenite and other tumor therapeutic agents. Therefore, we conclude that data in the current study have revealed a novel role for IKKβ in negatively regulating GADD45α protein stability and the contribution of p53-dependent IKKβ reduction to mediating cancer cell apoptosis.

## Introduction

GADD (growth arrest and DNA-damage inducible) 45α exert multiple functions in diverse cellular stress responses, including cell cycle arrest, cell senescence, apoptosis, DNA-damage repair and epigenetic modifications [1, 2]. The signaling cascades responsible for the induced expression of GADD45α are complex under various stress conditions and might involve different mechanisms, including transcriptional, posttranscriptional, translational and posttranslational events [3]. Our previous studies demonstrated that GADD45α undergoes constitutive ubiquitination and degradation in resting hepatoma cells. Arsenite exposure can block the ubiquitination and degradation of the endogenous GADD45α, leading to the accumulation of this protein and cellular apoptosis [4, 5]. We further identified that MDM2 is the E3 ubiquitin ligase for GADD45α. Arsenite exposure induces ribosomal stress responses, which result in the enhanced interaction between the ribosomal protein S7 and MDM2, and the interruption of MDM2-dependent GADD45α degradation [6]. These findings have thus provided a new model for GADD45α in mediating arsenite-induced hepatoma cell apoptosis through modulating its protein stability.

p53 is a transcriptional factor that can regulate the transcription of various downstream target genes and therefore plays multiple roles in growth arrest, DNA repair, senescence, autophagy, apoptosis, metabolism, development, and other processes [7]. p53 activates gene expression via binding to one of the specific p53 responsive elements (REs), consisting of two copies of a decamer motif separated by 0 to 13 bp of random nucleotide [8]. In addition to diverse p53-REs in the genome, multiple posttranslational modifications on p53 and the existence of binding partners for p53 also contribute substantially to the different affinity of p53 for the target promoters and the diversity of p53′s transcriptional events [9]. In addition to its well-known role in transcription activation, p53 has also been shown to repress the expression of a large number of targets [10–12]. The most commonly reported models for p53-dependent transcriptional repression involves the direct interaction of p53 with other transcriptional repressors to the target gene promoter or interference with the function of other transcriptional activators by p53 to mediate the inhibition of a target [13–19]. In other cases, genes repressed by p53 lack apparent p53 binding but involve physical interactions of p53 with other transcriptional activators (such as TBP, Sp-1, NF-Y, and Ets-1) and interfering with their promoter accessibility or transactivity [20–25].

IKKα and IKKβ, which are catalytic subunits of the I-κB kinase (IKK) complex, cooperatively mediate the activation of the transcriptional factor NF-κB and therefore play multiple roles under various conditions. Despite structural similarity, the mechanisms underlying the actions of IKKα and IKKβ in NF-κB activation are quite different [26, 27]. Moreover, both IKKα and IKKβ are demonstrated to possess some unique functions that are independent to NF-κB activity, but are mediated by NF-κB-unrelated subtracts, such as Aurora A, Maspin, 14-3-3σ, FOXO3, SMRT, p53, SRC3, c-Fos, p85α, mTOR, MDM2, and ATG16L1 [28–30]. Therefore, these findings have widened the understanding of the biological activities of IKKα and IKKβ, which act as multifunctional signaling proteins with roles going far beyond their well-known action in NF-κB pathway regulation.

In our previous reports, we demonstrated that both IKKα and IKKβ have the ability to mediate stress responses through NF-κB-independent mechanisms. Moreover, some specificity occurs between IKKα and IKKβ, because their substrates are exclusively regulated by one kinase but not the other [4, 31–33]. In the present study, we found that IKKβ, but not IKKα, negatively regulated GADD45α protein stability by stabilizing MDM2. In addition, the activation of the DAPK1/p53/Ets-1 signaling pathway exerted a transcriptional repression effect on *IKKβ*, which resulted in GADD45α accumulation and cell apoptosis under the exposure of tumor therapeutic agents, including arsenite.

## Results

### IKKβ reduction is required for mediating GADD45α accumulation and the pro-apoptotic response induced by arsenite

**Fig. 1:**
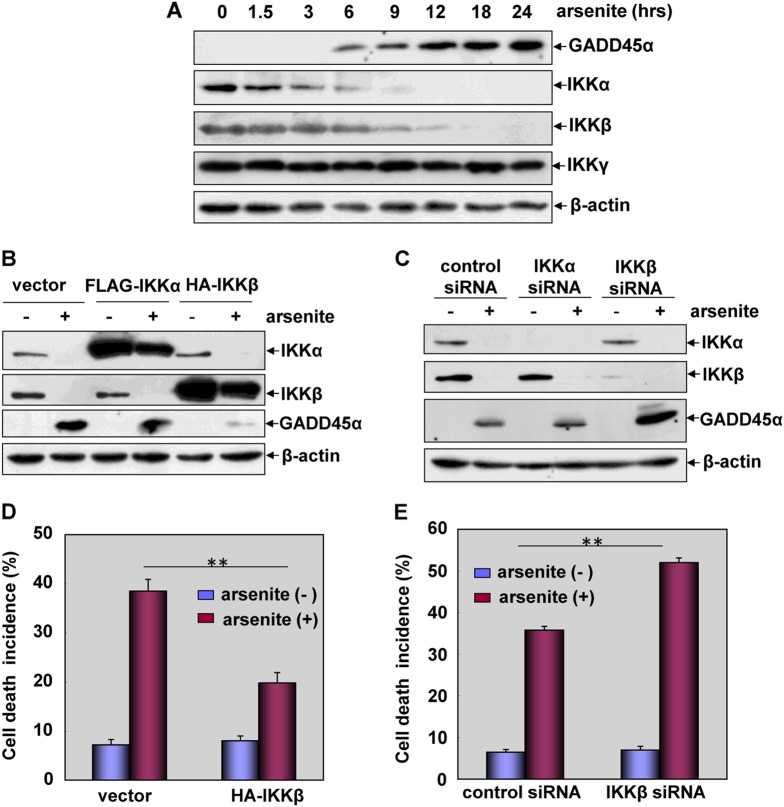
IKKβ reduction is required for mediating GADD45α accumulation and the pro-apoptotic response induced by arsenite exposure. **a** HepG2 cells were treated with arsenite (20 μM) for the indicated time periods and then the levels of GADD45α, IKKα, IKKβ and IKKγ were detected. **b** HepG2 cells were transfected with expression plasmids encoding FLAG-IKKα or HA-IKKβ or the control vector and then subjected to arsenite (20 μM) exposure. The expression levels of GADD45α, IKKα and IKKβ were detected at 12 h after arsenite treatment. **c** HepG2 cells were transfected with siRNA specifically targeting IKKα or IKKβ or the control siRNA and then treated as described in (**b**). The detections were also performed as described in (**b**). **d** HepG2 cells were transfected with HA-IKKβ construct or the control vector and then treated as described in (**b**). The cell death incidence was detected by flow cytometric assay at 24 h after arsenite exposure (**P < 0.01). **e** HepG2 cells were transfected with either IKKβ siRNA or the control siRNA and then treated as described in (**b**). The cell death incidence was detected at 24 h after arsenite exposure (**P < 0.01)

We previously revealed that GADD45α protein undergoes a constitutive degradation via the ubiquitin-proteasome pathway in resting HepG2 human hepatoma cells, which can be blocked by arsenite treatment, and then the accumulation of GADD45α contributes greatly to arsenite-induced cytotoxicity [5, 6]. In the current study, we found that time-dependent GADD45α accumulation was accompanied by downregulation of both IKKα and IKKβ, while the levels of the regulatory subunit of IKK, IKKγ, remained unchanged in the arsenite-treated HepG2 cells (Fig. 1a). Therefore, we investigated whether there was some functional link between the signaling events of GADD45α accumulation and IKKα/IKKβ downregulation in the arsenite-induced responses. As shown in Fig. 1b, c, compared with those in the control vector-transfected cells, GADD45α accumulation was dramatically inhibited in IKKβ, but not in IKKα-overexpressing cells. On the contrary, interrupting the expression of IKKβ, but not IKKα, significantly enhanced the accumulation of GADD45α. These results indicate that downregulation of IKKβ is critical for triggering GADD45α accumulation in arsenite-treated HepG2 cells.

In the following study, we observed that overexpression of IKKβ in HepG2 cells attenuated cell death incidence, while knockdown of IKKβ expression increased the percentage of apoptotic cells in response to arsenite stimulation (Fig. 1d, e). These results indicate that IKKβ functions as a protector in arsenite-induced pro-apoptotic responses by suppressing GADD45α expression; therefore, downregulation of IKKβ results in the induction of GADD45α accumulation and apoptosis in HepG2 cells.

### IKKβ reduces GADD45α protein stability by promoting its ubiquitination-dependent degradation

**Fig. 2:**
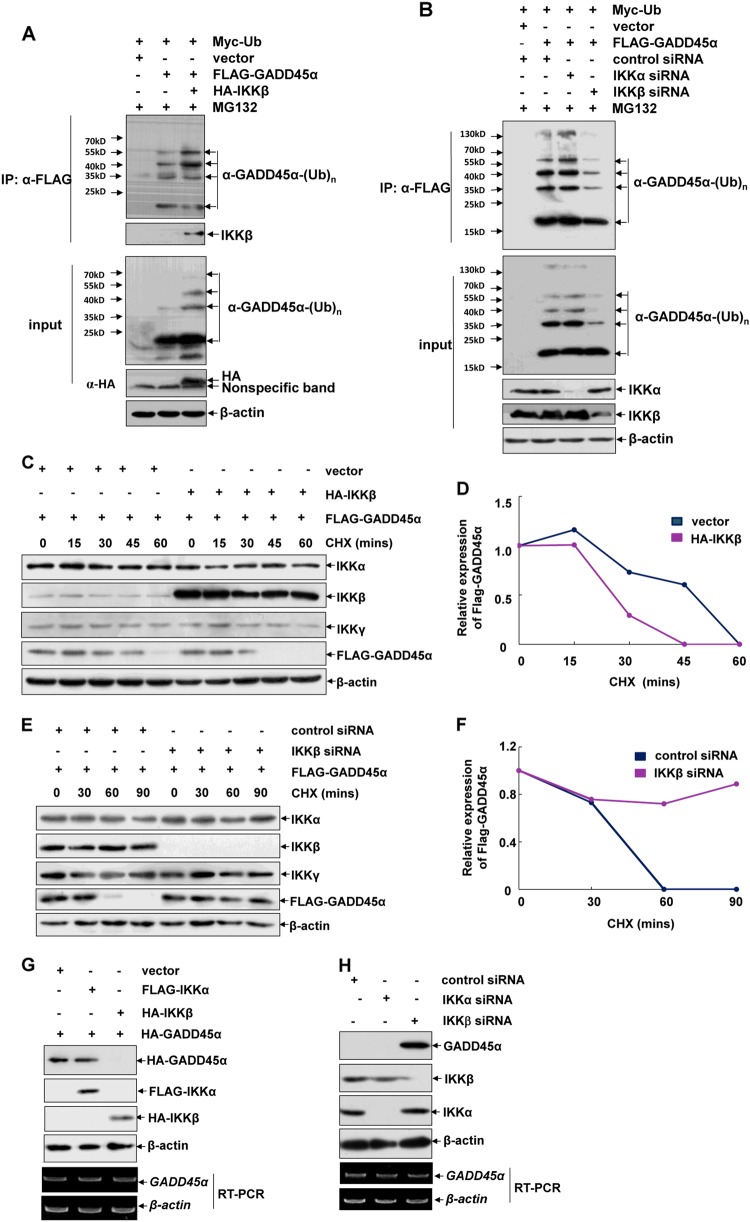
IKKβ reduces GADD45α protein stability by promoting its ubiquitination-dependent degradation. **a** HepG2 cells were transfected with Myc-Ub expression plasmid in combination with FLAG-GADD45α or HA-IKKβ constructs as indicated. Before harvesting, cells were treated with MG132 (10 μM) for 4 h. Then cell extracts were prepared and immunoprecipitated with anti-FLAG antibody. The ubiquitination of GADD45α was detected with anti-ubiquitin antibody. **b** HepG2 cells were transfected with Myc-Ub and FLAG-GADD45α expression plasmids in combination with siRNA specifically targeting IKKα or IKKβ or the control siRNA. Then the ubiquitination of GADD45α was detected as described in (**a**). **c** HepG2 cells were transfected with the expression plasmid encoding FLAG-GADD45α with or without the combination with the HA-IKKβ construct. Then the cells were subjected to CHX (10 μM) exposure as the indicated time periods, and the degradation of GADD45α was detected by anti-FLAG antibody. **d** The relative expression levels of GADD45α in (**c**) were quantified by Image-Pro Plus software. **e** HepG2 cells were transfected with the expression plasmid encoding FLAG-GADD45α with a combination of IKKβ siRNA or the control siRNA. Then the degradation of GADD45α was detected as described in (**c**). **f** The relative expression levels of GADD45α in (**e**) were quantified as described in (**d**). **g** HepG2 cells were transfected with HA-GADD45α expression plasmid in combination with FLAG-IKKα or HA-IKKβ constructs as indicated. Then the mRNA and protein levels of GADD45α were detected. **h** HepG2 cells were transfected with siRNA specifically targeting IKKα or IKKβ or the control siRNA. Then the mRNA and protein levels of GADD45α were detected. Cells were treated with MG132 (5 μM) for 30 min before harvesting

Arsenite-induced GADD45α accumulation results from the blockade of its constitutive ubiquitination-dependent degradation [6]. We next provided evidence that co-expression of HA-IKKβ enhanced poly-ubiquitination of GADD45α (Fig. 2a). By contrast, knockdown of endogenous IKKβ, but not IKKα, significantly decreased GADD45α ubiquitination levels (Fig. 2b). Consistently, co-expression of overdosed HA-IKKβ accelerated GADD45α degradation (Fig. 2c, d), while knockdown of endogenous IKKβ expression elongated the degradation dynamics of GADD45α (Fig. 2e, f). Moreover, overexpression of HA-IKKβ significantly reduced the ectopic GADD45α levels (Fig. 2g), while IKKβ depletion remarkably increased endogenous GADD45α protein stability (Fig. 2h). Most importantly, *GADD45α* mRNA transcription did not change under either IKKβ overexpression or depletion conditions (Fig. 2g, h). These data indicate that IKKβ possesses the novel function of mediating constitutive ubiquitination-dependent degradation of GADD45α, thereby decreasing cellular GADD45α protein stability in resting cells.

### IKKβ interacts with MDM2 and functions as an MDM2 co-activator under both steady state and arsenite exposure conditions

According to our previous report, MDM2 acts as the E3 ubiquitin ligase for GADD45α and triggers constitutive GADD45α ubiquitination and degradation, while the ribosomal protein S7 acts as a GADD45α stabilizer, which can suppress MDM2-dependent ubiquitination and degradation of GADD45α in both unstressed and arsenite-treated cells [6]. Therefore, we next focused on exploring whether the effect of IKKβ on reducing GADD45α stability is related to the aforementioned mechanism mediated by MDM2 and S7.

In HepG2 cells co-expressing HA-IKKβ and FLAG-S7, we did not find the signal indicating the interaction between IKKβ and S7. Moreover, the expression levels of FLAG-S7 remained the same with or without IKKβ overexpression (Fig. S1A). Consistently, no endogenous IKKβ-S7 complex formation was observed in both resting and arsenite-treated HepG2 cells (Fig. S1B). These data thus excludes the functional link between IKKβ and S7.

**Fig. 3:**
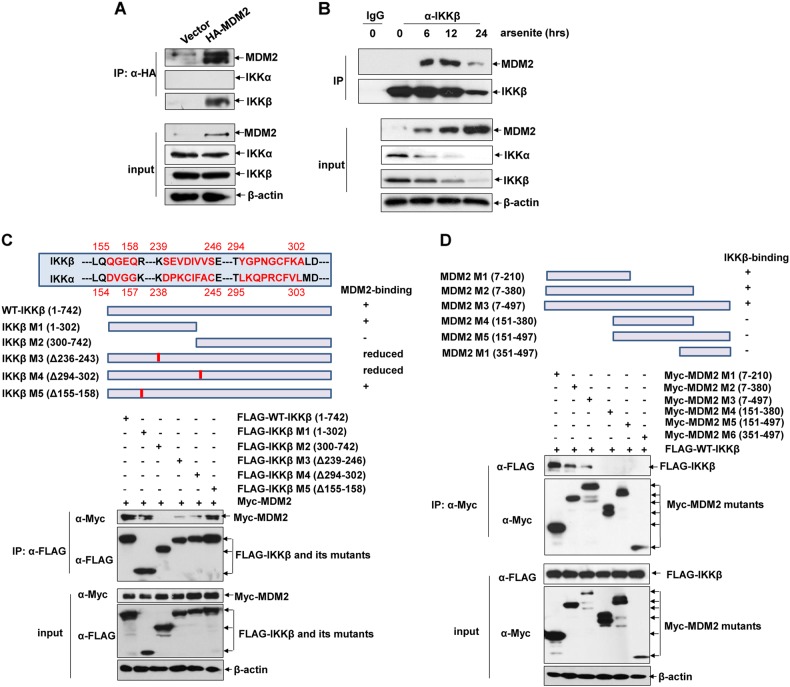
IKKβ interacts with MDM2 under both steady state and arsenite exposure conditions. **a** HepG2 cells were transfected with HA-MDM2 expression plasmid or the control vector. Cell lysate were immunoprecipitated with anti-HA antibody, and then the immunoprecipitants were probed with the antibodies as indicated. **b** HepG2 cells were treated with arsenite (20 μM) for the indicated time periods and then the interaction between endogenous MDM2 and IKKβ was detected by immunoprecipitation assay. **c** The three clusters of sequences in IKKβ (155–158, 239–246, and 294–302) showed large differences with the corresponding sequences in IKKα were indicated. Accordingly, IKKβ mutants were constructed and then their binding abilities to MDM2 were detected by co-IP. **d** MDM2 mutants were constructed as indicated and then their binding abilities to IKKβ were detected

Notably, after overexpression of HA-MDM2 in HepG2 cells, strong binding of MDM2 to endogenous IKKβ was readily observed, while no MDM2-associated IKKα signal was detected under the same conditions (Fig. 3a). In the following co-immunoprecipitation assay, we further observed the binding of endogenous IKKβ and MDM2 after a period of arsenite exposure, indicating that more endogenous IKKβ/MDM2 complexes were formed in response to arsenite stimulation. However, the enhancement interaction of IKKβ and MDM2 could only be transiently observed due to the reduction of IKKβ expression in response to long term of arsenite treatment (Fig. 3b).

Then, we attempted to map the binding domain involved in IKKβ/MDM2 complex formation. Based on the comparison of the amino acid sequences of IKKβ and IKKα, three clusters of sequences in IKKβ (155–158, 239–246 and 294–302) shows large differences with the corresponding sequences in IKKα (Fig. 3c). Therefore, IKKβ mutants, including or deleting these sequences, were constructed. Also shown in Fig. 3c, IKKβ mutants deleting the amino acids 239–246 and 294–302 showed a significant reduction in binding to MDM2, indicating that these sequences were involved in mediating IKKβ/MDM2 interaction. In the following study, we also identified that the amino acids 7–210 in MDM2 was responsible for interacting with IKKβ (Fig. 3d).

**Fig. 4:**
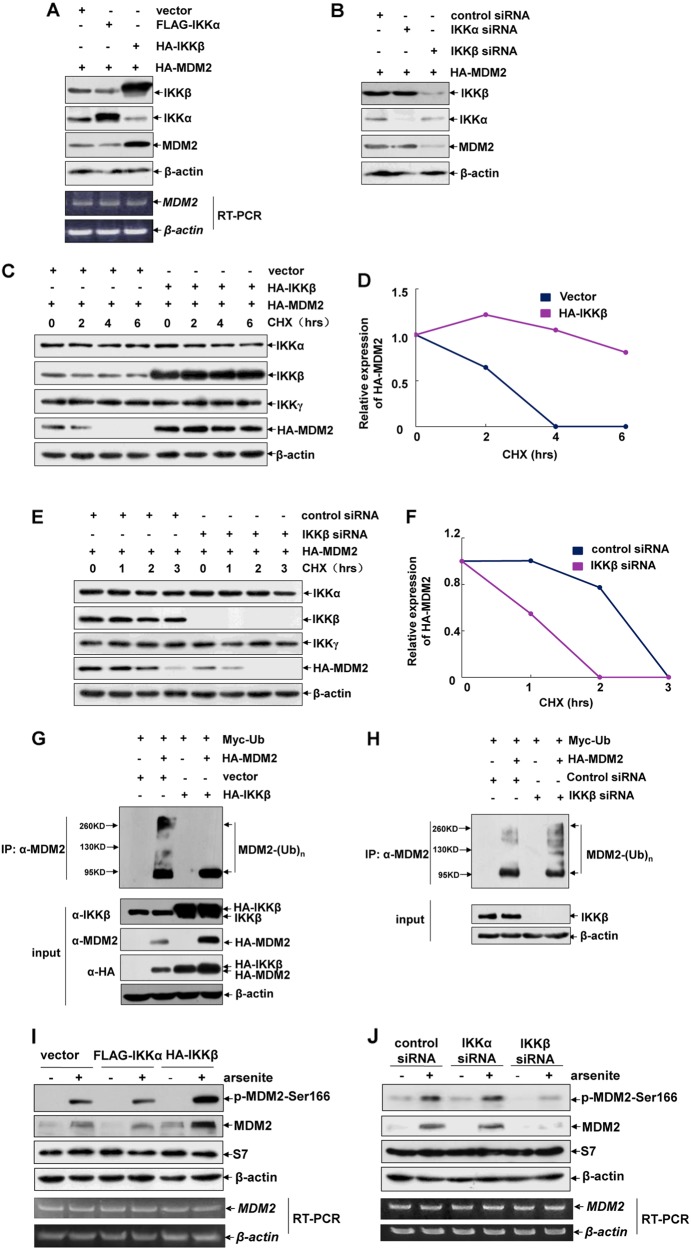
IKKβ functions as an MDM2 co-stabilizer under both steady state and arsenite exposure conditions. **a** HepG2 cells were transfected with HA-MDM2 expression plasmid in combination with FLAG-IKKα or HA-IKKβ constructs as indicated. Then the mRNA and protein levels of MDM2 were detected. **b** HepG2 cells were transfected with HA-MDM2 expression plasmid in combination with siRNA specifically targeting IKKα or IKKβ or the control siRNA. Then the mRNA and protein levels of MDM2 were detected. **c** HepG2 cells were transfected with the expression plasmid encoding HA-MDM2 with or without the combination of the HA-IKKβ construct. Then the cells were subjected to CHX (10 μM) exposure as the indicated time periods and the degradation of MDM2 was detected. **d** The relative expression levels of MDM2 in (**c**) were quantified as described in Fig. 2d. **e** HepG2 cells were transfected with the expression plasmid encoding HA-MDM2 with a combination of IKKβ siRNA or the control siRNA. Then the degradation of MDM2 was detected as described in (**c**). **f** The relative expression levels of MDM2 in (**e**) were quantified as described in Fig. 2d. **g** HepG2 cells were transfected with Myc-Ub expression plasmid in combination with HA-MDM2 or HA-IKKβ constructs as indicated. Cell extracts were prepared and immunoprecipitated with anti-MDM2 antibody. Then the ubiquitination of MDM2 was detected with anti-ubiquitin antibody. **h** HepG2 cells were transfected with Myc-Ub and HA-MDM2 expression plasmids in combination with IKKβ siRNA or the control siRNA. Then the ubiquitination of MDM2 was detected as described in (**g**). **i**–**j** HepG2 cells were transfected and treated as described in Fig. 1b, c. Then the phosphorylation and expression levels of MDM2 were detected at 12 h after arsenite exposure

Next, we analyzed whether IKKβ has the function to regulate MDM2 expression. As shown in Fig. 4a, b, introduction of IKKβ, but not IKKα, into HepG2 cells significantly increased ectopic MDM2 levels without affecting *MDM2* mRNA transcription, while interrupting the expression of endogenous IKKβ, but not IKKα, remarkably reduced the ectopic MDM2 levels. Consistently, degradation of HA-MDM2 was efficiently rescued by overexpression of IKKβ (Fig. 4c, d), while knockdown of IKKβ expression significantly accelerated HA-MDM2 degradation (Fig. 4e, f). In the following ubiquitination assay, we further observed the impairment of MDM2 ubiquitination in the IKKβ-overexpressed cells and enhancement of MDM2 ubiquitination by knocking down IKKβ expression (Fig. 4g, h). These data support that IKKβ inhibits MDM2 ubiquitination and stabilizes MDM2.

Because phosphorylation at Ser166 plays a critical role in regulating the protein stability of MDM2 [34], we next examined whether the function of IKKβ in stabilizing MDM2 involved any changes in MDM2-Ser166 phosphorylation. We observed remarkably increased MDM2 phosphorylation and accumulation by overexpression of IKKβ in the arsenite-treated HepG2 cells (Fig. 4i). By contrast, depletion of IKKβ expression almost totally blocked MDM2 phosphorylation and accumulation induced by arsentie (Fig. 4j). Under both conditions, *MDM2* mRNA transcription was not altered before and after arsenite exposure. Most importantly, up- or downregulation of IKKα expression did not affect the signaling events of MDM2 phosphorylation and accumulation (Fig. 4i, j). These data together indicate that IKKβ can interact with and increase the protein stability of MDM2 and therefore functions as an MDM2 co-activator to negatively regulate GADD45α accumulation under both resting and arsenite exposure conditions.

### The role of IKKβ in regulating MDM2 stability is unrelated to NF-κB transactivation but requires IKKβ kinase activity and IKKβ/MDM2 interaction

**Fig. 5:**
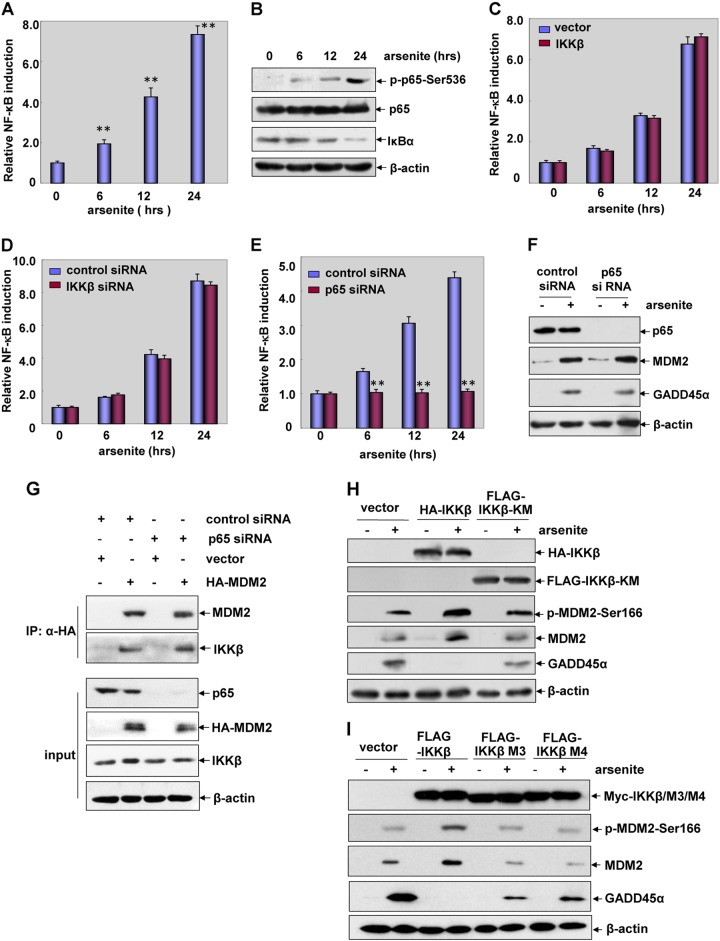
The role of IKKβ in regulating MDM2 and GADD45α protein stability is unrelated to NF-κB transactivation but requires IKKβ kinase activity and IKKβ/MDM2 interaction. **a** HepG2 cells were transfected with NF-κB-dependent luciferase reporter and the stable transfectants were established. The transfectants were exposed to arsenite (20 μM) for the indicated time period and then the induction of NF-κB-dependent luciferase activity was examined (**P < 0.01). **b** HepG2 cells were treated with arsenite (20 μM) for the indicated time periods and then phosphorylation of p65 and the levels of I-κB were detected. **c** HepG2 cells stably transfected with NF-κB-dependent luciferase reporter were transfected with HA-IKKβ construct or the control vector and then exposed to arsenite (20 μM). The induction of NF-κB-dependent luciferase activity was determined at the indicated time periods after arsenite exposure. **d**, **e** HepG2 cells stably transfected with NF-κB-dependent luciferase reporter were transfected with *IKKβ* siRNA (**d**) or *p65* siRNA (**e**) or their respective control siRNA and then exposed to arsenite (20 μM). The induction of NF-κB-dependent luciferase activity was determined at the indicated time periods after arsenite exposure. **f** HepG2 cells were transfected with *p65* siRNA or control siRNA and then exposed to arsenite (20 μM). The expression levels of GADD45α and MDM2 were detected at 12 h after arsenite exposure. **g** HepG2 cells were transfected with HA-MDM2 expression plasmid in combination with p65 siRNA or the control siRNA. Then the interaction of IKKβ and MDM2 was detected. **h** HepG2 cells were transfected with the expression plasmids encoding HA-IKKβ, FLAG-IKKβ-KM or the control vector. The phosphorylation and expression of MDM2 and the accumulation of GADD45α were detected at 12 h after arsenite exposure. **i** HepG2 cells were transfected with the expression plasmids encoding wild type IKKβ and its mutants M3/M4 with impaired MDM2 binding ability or the control vector. The detections were performed as described in (**h**)

We next determined whether the effect of IKKβ in regulating MDM2 and GADD45α protein stability is related to the activity of NF-κB. As shown in Fig. 5a, b, a significant upregulation of NF-κB-dependent luciferase activity, a time-dependent phosphorylation of p65 and degradation of I-κB were readily observed in HepG2 cells after arsenite treatment. Most importantly, the induction of NF-κB-dependent luciferase activities did not show detectable changes before and after IKKβ overexpression or depletion (Fig. 5c, d), indicating that arsenite induced NF-κB transactivation in HepG2 cells, which response was unrelated to IKKβ.

When p65 siRNA was transfected into HepG2 cells to inhibit NF-κB activation (Fig. 5e), no changes in the levels of MDM2 and GADD45α induction were detected in HepG2 cells treated with arsenite (Fig. 5f). Furthermore, the interaction of IKKβ and MDM2 was not affected under the same conditions (Fig. 5g). These data together indicate that the role for IKKβ in regulating MDM2 and GADD45α protein stability is unrelated to the activation status of NF-κB.

To address whether IKKβ kinase activity is required for regulating MDM2 and GADD45α induction in the arsenite response, wild type IKKβ or its kinase mutant, IKKβ-KM, was transfected into HepG2 cells. We found that overexpression of IKKβ-KM failed to enhance MDM2 phosphorylation and induction as the wild type IKKβ did, but efficiently rescued GADD45α accumulation under the same arsenite exposure conditions (Fig. 5h). Therefore, we conclude that IKKβ kinase activity is required for regulating MDM2 and GADD45α protein stability in response to arsenite stimulation. When the IKKβ mutants with reducing MDM2 binding ability (IKKβΔ239–246 and IKKβΔ294–302) were introduced into HepG2 cells, we observed similar responses as those obtained in the IKKβ-KM-overexpressing cells (Fig. 5i), indicating that the interaction of IKKβ and MDM2 is also required for enhancing MDM2 stability and GADD45α accumulation induced by arsenite.

### p53 links its downstream target Ets-1 to mediate transcriptional inhibition of *IKKβ* and GADD45α accumulation induced by arsenite

**Fig. 6:**
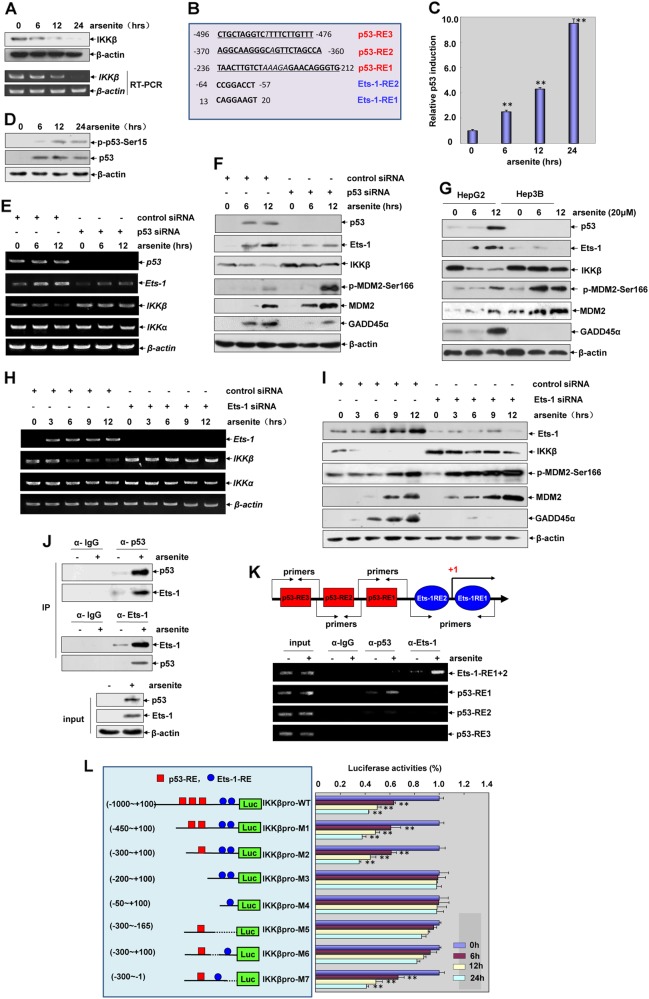
p53 links its downstream target Ets-1 to mediate transcriptional inhibition of *IKKβ* and GADD45α accumulation induced by arsenite. **a** HepG2 cells were treated with arsenite (20 μM) for the indicated time periods and then mRNA and protein expression levels of IKKβ were detected. **b** Analysis of the human *IKKβ* promoter (−1000 bp~ + 100 bp) indicated three putative p53-responsive elements (p53-RE1, 2, 3) containing 5, 1, and 1-nucleotide (italics) spacers, respectively, between the two decamer “half-sites” (bold). The potential putative Ets-1-responsive elements (Ets-1-RE1, 2) were also indicated. **c** HepG2 cells were transfected with a p53-dependent luciferase reporter and the stable transfectants were established. The transfectants were exposed to arsenite (20 μM) for the indicated time periods and then the induction of p53-dependent luciferase activity was examined (**P < 0.01). **d** HepG2 cells were treated with arsenite (20 μM) for the indicated time periods and then phosphorylation and accumulation of p53 were detected. (**e, f**) HepG2 cells were transfected with *p53* siRNA or control siRNA and then exposed to arsenite (20 μM). The mRNA and protein levels of IKKβ and Ets-1, phosphorylation and expression of MDM2, and the accumulation of GADD45α were detected at 12 h after arsenite exposure. **g** HepG2 and Hep3B (p53-/-) cells were treated with arsenite (20 μM) for the indicated time periods and then the detections were performed as described in (**f**). **h**, **i** HepG2 cells were transfected with Ets-1 siRNA or its control siRNA and then exposed to arsenite (20 μM). Then the detections were performed as described in (**e** and **f**). **j** HepG2 cells were left untreated or treated with arsenite (20 μM). Cell lysate were immunoprecipitated with anti-p53, anti-Ets-1 antibody or the control IgG, and then the immunoprecipitants were probed with the antibodies as indicated. (**k**) HepG2 cells were left untreated or treated with arsenite (20 μM). The soluble chromatin was prepared and then ChIP assay was performed to detect the binding ability of p53 and Ets-1 within the human *IKKβ* promoters. **l** A summary of the constructed luciferase reporter driven by the wild type and mutated *IKKβ* promoter. The three putative p53-REs (■) and two putative Ets-1-REs (●) are indicated. HepG2 cells were transfected with the wild type and mutated *IKKβ* luciferase reporters and then exposed to arsenite (20 μM). The luciferase activities were detected at 24 h after arsenite exposure (**P < 0.01)

Next, we focused on investigating the upstream signaling events involved in the suppression of IKKβ expression. Here we found that downregulation of IKKβ expression in HepG2 cells was accompanied by a reduction in *IKKβ* mRNA levels after arsenite exposure (Fig. 6a), indicating that arsenite stimulation repressed the transcription of *IKKβ*. Therefore, an analysis of the −1000 bp~ + 100 bp region of the human *IKKβ* promoter was performed, and DNA sequences perfectly or partially matched to the binding sites for p53 and Ets-1 were identified (Fig. 6b), thus raising the hypothesis that p53 and Ets-1 might be implicated in arsenite-induced transcriptional repression of *IKKβ*. As shown in Fig. 6c, d, time-dependent enhancement of p53-driven luciferase activities and upregulation of p53 phosphorylation at Ser15 were readily observed, indicating p53 transactivation under arsenite exposure. When p53 expression was suppressed, reduction in *IKKβ* mRNA transcription as well as its protein synthesis was efficiently rescued. Moreover, a remarkable enhancement of MDM2 phosphorylation and induction and a significant inhibition of GADD45α accumulation were also detected under the same conditions (Fig. 6e, f). The data obtained in the p53-/- hepatoma cells (Hep3B) further confirmed the above results detected in the p53 siRNA-transfected HepG2 cells (Fig. 6g). Together, these data indicate that p53 transactivation is responsible for the transcriptional repression of *IKKβ* and then results in the accumulation of GADD45α in the arsenite responses.

Additionally, as shown in Fig. 6e–g, efficient inhibition of Ets-1induction was observed in both p53 siRNA-transfected HepG2 cells and p53−/− Hep3B cells, indicating that Ets-1 is a transcriptional target of p53 under arsenite exposure. When Ets-1 expression was suppressed, we observed rescue of IKKβ repression, upregulation of MDM2 phosphorylation and induction and the block of GADD45α accumulation in HepG2 cells (Fig. 6h, i). Moreover, data from the co-immunoprecipitation assay further showed a strong interaction between p53 and Ets-1 after arsenite exposure (Fig. 6j). These data together indicate that p53 and Ets-1 cooperatively mediate the repression of *IKKβ* transcription and induction of GADD45α expression in the arsenite responses.

In the following ChIP assay, we observed that among the three motifs homologous to p53-responsive elements within the human *IKKβ* promoter, p53 could recruit only to the proximal site (p53-RE1), upon arsenite exposure. Additionally, a strong association of Ets-1 with the chromatin regions containing two Ets-1-REs was readily observed (Fig. 6k). These data indicate that the p53/Ets-1 complex mediates the transcriptional repression effect by recruiting to the adjacent DNA responsive elements within the *IKKβ* promoter.

To further confirm the above results, a luciferase assay was performed by using the constructed luciferase reporters driven by the wild type, deletion mutant, or point mutant of the *IKKβ* promoter. As shown in Fig. 6l, arsenite exposure induced a time-dependent reduction of wild type *IKKβ* promoter-driven luciferase activity, which response was not altered by deletion of the distal p53-RE3 and p53-RE2 (M1 and M2), but was totally interrupted by proximal p53-RE1 deletion (M3 and M4) within the *IKKβ* promoter. However, the *IKKβ* promoter mutant containing only p53-RE1 but neither of the Ets-1-REs (M5) did not respond to arsenite stimulation, while the mutated *IKKβ* promoter containing p53-RE1 and Ets-1-RE2 (M7) showed a significant reduction in the luciferase activity similar to the wild type, which response was totally lost when Ets-1-RE2 was replaced by Ets-1-RE1 (M6). Taken together, p53-RE1 and Ets-1-RE2 function as the DNA responsive elements in mediating the synergistic effect of p53 and Ets-1 on the suppression of *IKKβ* transcription.

### DAPK1 is responsible for mediating p53/Ets-1 pathway activation and subsequent IKKβ reduction-dependent GADD45α accumulation induced by arsenite

**Fig. 7:**
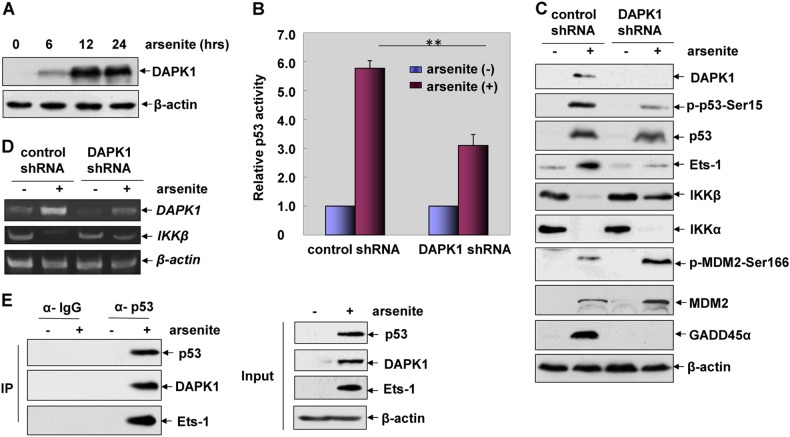
DAPK1 is responsible for mediating p53/Ets-1 pathway activation and the subsequent IKKβ reduction-dependent GADD45α accumulation induced by arsenite. **a** HepG2 cells were treated with arsenite (20 μM) for the indicated time periods and then the expression level of DAPK1 was detected. **b** HepG2 cells stably transfected with p53-dependent luciferase reporter were transfected with DAPK1 shRNA or the control shRNA and then exposed to arsenite (20 μM). The induction of p53-dependent luciferase activity was determined at 24 h after arsenite exposure. **c**, **d** HepG2 cells were transfected with DAPK1 shRNA or the control shRNA and then exposed to arsenite (20 μM). The induction of p53/Ets-1/IKKβ/MDM2/ GADD45α pathway activation was determined at 12 h after arsenite exposure. **e** HepG2 cells were left untreated or treated with arsenite (20 μM). Cell lysate were immunoprecipitated with anti-p53 antibody or the control IgG, and then the immunoprecipitants were probed with the antibodies as indicated

In the following study to explore the possible upstream protein kinase (such as ATR, CHK1, LKB1, DAPK1) that might be responsible for triggering p53-dependent IKKβ repression induced by arsenite, we found the significant upregulation of DAPK1 expression (Fig. 7a) and activation of ATR, CHK1 and LKB1 (data not shown) in HepG2 cells after arsenite exposure. Although all of the above protein kinases were involved in p53 transactivation (Fig. 7b, c and data not shown), only DAPK1 was shown to have the ability to regulate the induction of Ets-1 and the repression of *IKKβ*, evidenced by the inhibition of Ets-1 induction and rescue of IKKβ reduction with the impairment of DAPK1 expression (Fig. 7c, d). Moreover, arsenite stimulation triggered the interaction of DAPK1 with the p53/Ets-1 complex (Fig. 7e). Additionally, interrupting DPAK1 induction enhanced MDM2 levels and inhibited GADD45α accumulation in the HepG2 cells exposed to arsenite (Fig. 7c). These data together indicate the novel mechanism of DAPK1 in mediating p53 transactivation and the subsequent signal transduction of the Ets-1/IKKβ/MDM2/GADD45α cascade induced by arsenite.

### The universal role of the DAPK1/p53/Ets-1/IKKβ/MDM2/GADD45α cascade activation in mediating the cytotoxic effects of tumor therapeutic agents

**Fig. 8:**
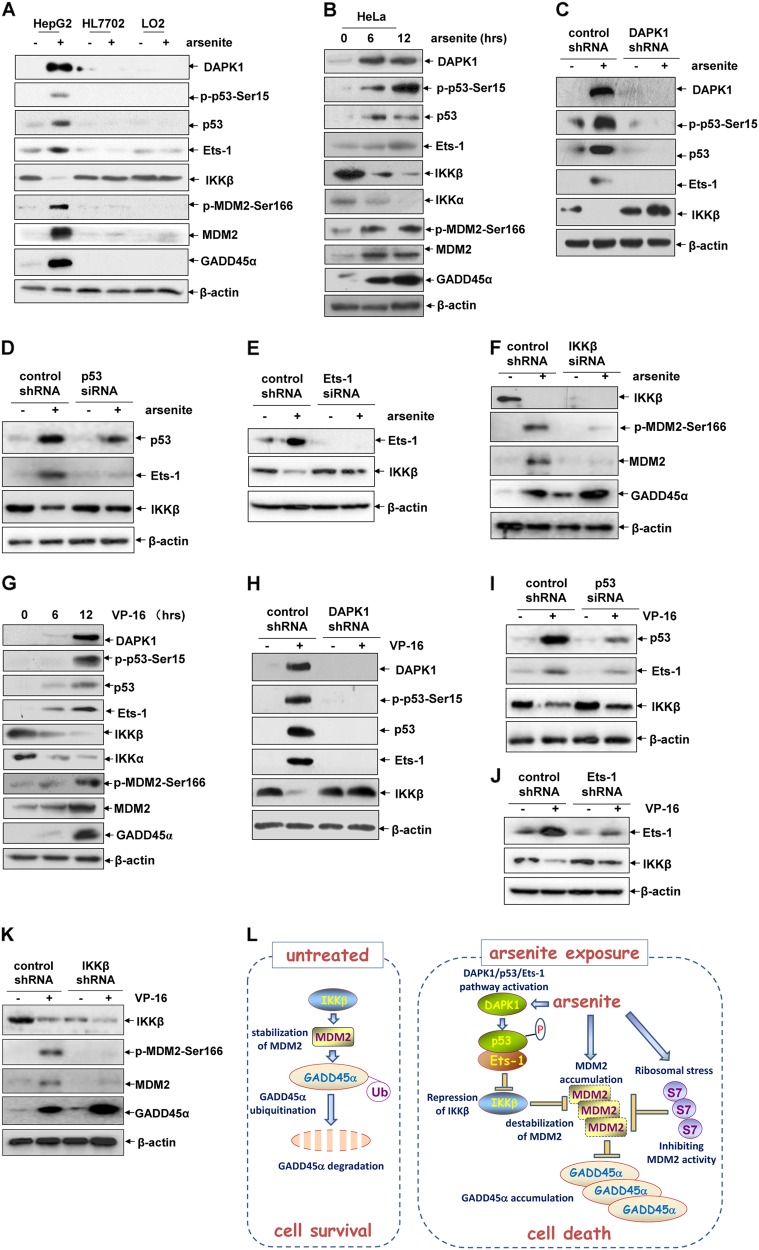
The universal role of the DAPK1/p53/Ets-1/IKKβ/MDM2/GADD45α cascade activation in mediating the cytotoxic effects of tumor therapeutic agents. **a** HepG2, HL7702, and LO2 cells were left untreated or treated with arsenite (20 μM). Then the activation of the DAPK1/p53/Ets-1/IKKβ/MDM2/GADD45α pathway was detected at 12 h after arsenite exposure. **b** HeLa cells were left untreated or treated with arsenite (20 μM) for the indicated time periods and then the detections were performed as described in (**a**). **c**–**e** HeLa cells were transfected with DAPK1 shRNA, p53 siRNA, Ets-1 siRNA or their control si/sh RNAs followed by treatment with arsenite (20 μM). Then the expression levels of IKKβ were detected. **f** Hela cells were transfected with IKKβ siRNA or its control siRNA followed by treatment of arsenite (20 μM). Then the expression levels of MDM2 and GADD45α were detected. **g** HepG2 cells were left untreated or treated with VP-16 (25 μM) for the indicated time periods and then the detections were performed as described in (**a**). **h**–**j** HepG2 cells were transfected and treated as described in (**c**-**e**). Then the expression levels of IKKβ were detected. **k** HepG2 cells were transfected and treated as described in (**f**). Then the expression levels of MDM2 and GADD45α were detected. **l** Working model of DAPK1/p53/Ets-1/IKKβ/MDM2/GADD45α and S7/MDM2/GADD45α pathway activation in mediating the apoptotic responses induced by arsenite. In the resting cells, constitutive expression of IKKβ functioned as the stabilizer of MDM2, which triggered the ubiquitination of degradation of GADD45α. Arsenite exposure induced the activation of the DAPK1/p53/Ets-1 pathway, which resulted in the transcriptional inhibition of IKKβ and destabilization of MDM2, therefore impaired ubiquitination of degradation of GADD45α and leading to GADD45α accumulation-dependent cell death. Arsenite exposure also induced ribosomal stress and results in the inhibition of MDM2 activity by S7, which also contributes to GADD45α accumulation-dependent cell death (Gao et al. 2013)

In the following study, we found that compared with the strong activation of the DAPK1/p53/Ets-1/IKKβ/MDM2/GADD45α pathway in arsenite-treated HepG2 cells, no signals indicating the activation of any of the components in this pathway were observed in either of the two normal human diploid hepatic cells, HL7702 and LO2, which are insensitive to arsentie exposure, as shown in our previous study (Fig. 8a) [5]. This result indicates the specific role of the DAPK1/p53/Ets-1/IKKβ/MDM2/GADD45α cascade activation in mediating the cytotoxic effect of arsenite in hepatoma cells.

Then, we observed the activation of the DAPK1/p53/Ets-1/IKKβ/MDM2/GADD45α pathway in a variety of other arsenite-sensitive cancer cell lines, including HeLa (Fig. 8b), MCF7, HCT116 and A549 (Fig. S2). Moreover, knocking down DAPK1, p53 or Ets-1 expression almost totally recovered IKKβ reduction induced by arsenite in HeLa cells (Fig. 8c–e). In addition, nterrupting IKKβ expression inhibited MDM2 induction and increased GADD45α accumulation (Fig. 8f). These data indicate that activation of the DAPK1/p53/Ets-1/IKKβ/MDM2/GADD45α cascade might be a universal event in mediating the cytotoxic effects of arsenite in different cancer cells.

Next, we found that the DAPK1/p53/Ets-1/IKKβ/MDM2/GADD45α cascade was also active in HepG2 cells treated with other tumor therapeutic agents, such as VP-16 and 5-Fu (Fig. 8g and Fig. S3). Most importantly, we also observed the block of IKKβ downregulation with the impairment of DAPK1, p53 and Ets-1 induction, the reduced MDM2 phosphorylation and induction, and enhanced GADD45α accumulation by knocking down IKKβ expression in the VP-16-treated HepG2 cells (Fig. 8h–k). Therefore we conclude that the DAPK1/p53/Ets-1/IKKβ/MDM2/GADD45α pathway might have the potential to deliver the pro-apoptotic effects induced by certain tumor therapeutic agents.

## Discussion

The studies of disclosing the signaling events responsible for the induced expression of GADD45α have attracted wide attention due to the critical role of GADD45α in many important cellular functions [3]. The contribution of our group in this field is to disclose the novel posttranslational mechanism involved in regulating GADD45α protein stability under both resting and arsenite exposure conditions, which is mediated by the ribosomal protein S7 and the E3 ubiquitin ligase MDM2 [4–6]. In the present study, we further disclose the novel role of IKKβ in regulating MDM2-dependent GADD45α ubiquitination and degradation, thus providing a new mechanism involved in the regulation of GADD45α protein stability. However, to be different from our previous report, which demonstrated no involvement of p53 in regulating S7-dependent GADD45α induction [6], the role of IKKβ in regulating GADD45α stability is unrelated to S7 but mediates by p53. For another discrepancy, S7 enters for GADD45α regulation via binding MDM2 and blocking its activity to trigger the ubiquitination and degradation of GADD45α, in which process the MDM2 level is not altered by S7 [6]. By contrast; p53-dependent IKKβ reduction is linked to GADD45α regulation by decreasing MDM2 levels. Together with our previous reports, we have disclosed both S7-dependent/p53-independent and p53-dependent/S7-independent posttranslational mechanisms in regulating GADD45α expression by targeting MDM2 activity and stability, respectively (Fig. 8l).

The specificity of the two catalytic subunits of IKK in regulating signaling events unrelated to NF-κB has been revealed in depth by a number of studies, including our own [28–33, 35]. In the current study, we further elucidates the role of IKKβ implicated in MDM2 protein stability under both resting and arsenite exposure conditions, an effect that is unrelated to IKKα and NF-κB activity. In fact, the IKKβ-dependent upregulation of MDM2 expression has been reported in a previous study, which demonstrates that the expression of MDM2 is regulated at the transcriptional level by IKKβ and canonical NF-κB pathway-dependent manner [36]. However, in contrast to that report; here we found that IKKβ regulated MDM2 phosphorylation and stability without affecting *MDM2* mRNA transcription (Fig. 4a, i, j). Moreover, the function of IKKβ involved in the posttranslational modification of MDM2 depended largely on the specific binding between these two signaling molecules (Figs. 3 and 5i). According to the primary sequences analysis on IKKα and IKKβ, the amino acids 239–246 and 294–302 in IKKβ involving in MDM2 interaction are not matched to the corresponding sequences in IKKα (amino acids 238–245, 295–303) (Fig. 3c). These sequence differences might be the reason why these two IKK subunits expressed different binding ability to MDM2.

Notably, both IKKα and IKKβ expression levels were downregulated under the exposure of arsenite and other tumor therapeutic agents (Figs. 1a, 8b, g), but only a reduction of IKKβ was triggered by p53-dependent transcriptional repression. Therefore, it is interesting to disclose why p53 selectively represses the transcription of *IKKβ* in the cytotoxic responses. As previously described, the promoter selectivity of p53 in the transcriptional program is determined by locus-specific cis-regulatory elements, posttranslational modifications of p53, and the covalent and noncovalent p53 binding partners [10, 11]. p53-RE is originally defined as RRRCWWGYYY (*n* = 0–13) RRRCWWGYYY (where R is adenine or guanine, W is a purine base, and Y is a pyrimidine base). Any subtle differences in the sequence or spacing of the p53-binding sites at different genes could result in changes in the DNA secondary structure or in the topology of the chromatin, thereby altering the affinity of p53 for the target promoters [11]. In fact, the putative p53-REs were identified within both the *IKKα* [20] and *IKKβ* (Fig. 6b) promoter regions. However, only recruitment of the activated p53 to p53-RE1 within the *IKKβ* promoter was observed in HepG2 cells after arsenite exposure (Fig. 6k); no *IKKα* promoter chromatin-associated p53 was detected under the same arsenite exposure conditions (data not shown). Therefore, the difference between the affinity of p53 for the different architecture of p53-REs within the *IKKα* and *IKKβ* promoter may play a critical role in determining the selective transcriptional repression of *IKKβ*. For another reason, p53/Ets-1 complex formation and the binding of Ets-1 to Ets-1-RE2 within the *IKKβ* promoter were also shown to be required for p53 to deliver the transcriptional repression effect (Fig. 6l). Based on these results, we believed that Ets-1, which functions as a downstream target and the binding partner of p53, also contributes to regulating the ability of p53 in recognizing the specific p53-RE within the *IKKβ* promoter.

In summary, we have revealed the novel mechanism of IKKβ in regulating GADD45α stability and the previously unidentified selective transcriptional repression of *IKKβ* by p53 signaling. Therefore, these findings have provided novel evidence for the cross-talk between p53, IKK and GADD45α under stress conditions.

## Materials and methods

### Plasmids, antibodies, and reagents

The plasmids expressing FLAG-GADD45α, FLAG-S7, HA-MDM2, Myc-Ub, FLAG-IKKα, HA-IKKβ, FLAG-IKKβ-KM, p53-Luc, and NF-κB-Luc were described in our previous reports [6, 31–33, 37, 38]. Human DAPK1 shRNA was constructed by using the GeneSuppressor System (Imgenex). The siRNAs targeting IKKα, IKKβ, p53, Ets-1, and the primary antibodies against IKKα (2682), IKKβ (2370), myc (2276), ubiquitin (2933), DAPK1 (3008), p53 (2524), p-p53-Ser15 (9284) and Ets-1 (14096) were purchased from Cell Signaling Technology (Beverly, MA, USA). The primary antibodies against IKKγ (sc-8330), GADD45α (sc-797), S7 (sc-100834) and HA (sc-7392) were obtained from Santa Cruz Biotechnology (Santa Cruz, CA, USA). The antibody against MDM2 (OP46) was purchased from Calbiochem (Darmstad, Germany) and Anti-FLAG antibody (M2), MG132, cyclohexamide (CHX) and arsenite were purchased from Sigma (St. Louis, MO, USA).

### Generation of human *IKKβ* promoter deletion and mutation constructs

In addition to the plasmid containing wild type human *IKKβ* promoter, various 5′-3′ or 3′-5′ deletion constructs and site-directed *IKKβ* promoter mutants were made by regular RT-PCR or by using in vitro site-directed mutagenesis system (Promega). The *IKKβ* promoter sequence containing three putative p53-responsive elements (p53-RE1/2/3) and two putative Ets-1-responsive elements (Ets-1-RE/2) (WT), deletion mutant of p53-RE3 (M1), deletion mutant of p53-RE3 and p53-RE2 (M2), deletion mutant of all three p53-REs (M3), deletion mutant of all three p53-REs and the distal Ets-1-RE2 (M4), the mutant only containing the proximal p53-RE1 (M5), the mutant containing p53-RE1 and the proximal Ets-1-RE1 (M6) and the mutant containing p53-RE1 and the distal Ets-1-RE2 (M7) were respectively obtained and then inserted into the pGL3 basic vector to construct the different *IKKβ* promoter-driven luciferase reporter plasmids.

### Cell culture and transfection

HepG2, HeLa, MCF7, A549, Hep3B, HCT116, HL7702, and LO2 cells were maintained in DMEM supplement with 10% fetal bovine serum, 1% penicillin and streptomycin. Mycoplasma contamination were tested and excluded. Cells were transfected using LipofectAMINE 2000 or LipofectAMINE^TM^RNAi MAX (Invitrogen) according to the manufacturer’s instructions.

### Luciferase reporter assay

p53-Luc and NF-κB-Luc were transfected into HepG2 cells and then the stable transfectants were established. Wild type and the mutated *IKKβ* promoter-driven luciferase reporter plasmids were transiently transfected into HepG2 cells in combination of the internal control reporter. The stable transfectants and the HepG2 cells transiently transfected with *IKKβ* promoter-driven luciferase reporter plasmids were exposed to asenite for 36 h, and then the luciferase activity was tested as previously described [37]. Triplicate samples were used in the detections and at least three independent experiments were performed.

### RNA isolation and RT-PCR assay

Trizol reagent was used to extract total RNA and then RT-PCR was performed as previously described [37]. To analyze the transcription of *IKKα*, *IKKβ*, *p53*, *ETS-1, MDM2, DAPK1*, the specific primers (can be obtained if required) were designed to amplify the target cDNAs.

### ChIP assay

ChIP assay was performed as described in our previous report [35]. ChIP primers were designed and synthesized to specifically amplify the regions covering the putative p53-REs and/or Ets1-REs within the human *IKKβ* promoter. The sequences of the primers can be available by requesting.

### Immunoprecipitation and immunoblot assay

HepG2 cells were transfected with various combinations of the expression plasmids encoding wild type MDM2, IKKβ or their mutants. Then reciprocal immunoprecipitations (IPs) were performed to detect the binding domains involved in MDM2/IKKβ interaction. HepG2 cells stimulated with arsenite and then the complex formation for the endogenous MDM2/IKKβ, p53/Ets-1, DAPK1/p53/Ets-1 were determined by immunoprecipitation. Cellular protein preparation and immunoblot assays were performed as described previously [5, 6].

### In vivo ubiquitination assay

Myc-Ub and FLAG-GADD45α were transfected into HepG2 cells in the absence or presence of the constructs encoding IKKβ or of the specific siRNAs targeting IKKα or IKKβ. The ubiquitination of GADD45α was detected as described in our previous report [6].

### Immunofluorescence assay

To detect the subcellular distribution of MDM2 and IKKβ, HepG2 cells with or without arsenite exposure were fixed and then incubated with the primary antibodies against MDM2 or IKKβ and the FITC or PE-conjugated secondary antibodies. The signal was monitored using the confocal microscopy (ZEISS, LSM510 META).

### Cell apoptosis assay

Arsenite-induced apoptosis in HepG2 cells was determined according to the percentage of propidium iodide (PI)-positive cells as described in our previous reprots [5, 6].

### Statistics

To determine the effect of a single treatment within a group, Student’s t-test was used to test the significance of the data. To determine the effects of treatment × group interactions, factorial design (ANOVA) was employed to test the significance of the data. At least three independent experiments were performed. The results were presented as the mean ± SD. The level of significance was set at *P* < 0.05.

## Electronic supplementary material

supplemented data

## References

[1] Tamura RE, de Vasconcellos JF, Sarkar D, Libermann TA, Fisher PB, Zerbini LF (2012). GADD45α proteins: central players in tumorigenesis. Curr Mol Med.

[2] Moskalev AA, Smit-McBride Z, Shaposhnikov MV, Plyusnina EN, Zhavoronkov A, Budovsky A (2012). Gadd45 proteins: relevance to aging, longevity and age-related pathologies. Ageing Res Rev.

[3] Gao M, Guo N, Huang CS, Song L (2009). Diverse roles of GADD45α in stress signaling. Curr Protein Pep Sci.

[4] Song L, Li JX, Zhang DY, Liu ZG, Ye JP, Zhan QM (2006). IKKβ programs to turn on the GADD45α-MKK4-JNK apoptotic cascade specifically via p50 NF-κB in arsenite response. J Cell Biol.

[5] Gao M, Dong W, Hu MR, Yu M, Guo L, Qian L (2010). GADD45α mediates arsenite-induced cell apoptotic effect in human hepatoma cells via JNKs/AP-1-dependent pathway. J Cell Biochem.

[6] Gao M, Li XG, Dong W, Jin R, Ma HH, Yang PX (2013). Ribosomal protein S7 regulates arsenite-induced GADD45α expression by attenuating MDM2-mediated GADD45α ubiquitination and degradation. Nucleic Acids Res.

[7] Vousden KH, Prives C (2009). Blinded by the light: the growing complexity of p53. Cell.

[8] Fischer M (2017). Census and evaluation of p53 target genes. Oncogene.

[9] Sullivan KD, Gallant-Behm CL, Henry RE, Fraikin JL, Espinosa JM (2012). The p53 circuit board. Biochim Biophys Acta.

[10] Beckerman R, Prives C (2010). Transcriptional regulation by p53. Cold Spring Harb Perspect Biol.

[11] Sullivan KD, Galbraith MD, Andrysik Z, Espinosa JM (2018). Mechanisms of transcriptional regulation by p53. Cell Death Differ.

[12] Ho J, Benchimol S (2013). Transcriptional repression mediated by the p53 tumoursuppressor. Cell Death Differ.

[13] Godar S, Ince TA, Bell GW, Feldser D, Donaher JL, Bergh J (2008). Growth-inhibitory and tumor-suppressive functions of p53 depend on its repression of CD44 expression. Cell.

[14] Ho JS, Ma W, Mao DY, Benchimol S (2005). p53-dependent transcriptional repression of c-myc is required for G1 cell cycle arrest. Mol Cell Biol.

[15] Lin TX, Chao C, Saito S, Mazur SJ, Murphy ME, Appella E (2005). p53 induces differentiation of mouse embryonic stem cells by suppressing *Nanog* expression. Nat Cell Biol.

[16] Zhang Y, Wang JS, Chen LL, Zhang Y, Cheng XK, Heng FY (2004). Repression of hsp90β gene by p53 in UV irradiation-induced apoptosis of Jurkat cells. J Biol Chem.

[17] Hoffman WH, Biade S, Zilfou JT, Chen JD, Murphy M (2002). Transcriptional repression of the anti-apoptotic *survivin* gene by wild type p53. J Biol Chem.

[18] St Clair S, Giono L, Varmeh-Ziaie S, Resnick-Silverman L, Liu WJ, Padi A (2004). DNA damage-induced downregulation of Cdc25C is mediated by p53 via two independent mechanisms: one involves direct binding to the cdc25C promoter. Mol Cell.

[19] Li B, Lee MY (2001). Transcriptional regulation of the human DNA polymerase δ catalytic subunit gene POLD1 by p53 tumor suppressor and Sp1. J Biol Chem.

[20] Gu LB, Zhu NX, Findley HW, Woods WG, Zhou MX (2004). Identification and characterization of the IKKα promoter. J Biol Chem.

[21] Kim E, Gunther W, Yoshizato K, Meissner H, Zapf S, Nusing RM (2003). Tumor suppressor p53 inhibits transcriptional activation of invasion gene thromboxane synthase mediated by the proto-oncogenic factor ets-1. Oncogene.

[22] Pastorcic M, Das HK (2000). Regulation of transcription of the human presenilin-1 gene by ets transcription factors and the p53 protooncogene. J Biol Chem.

[23] Imbriano C, Gurtner A, Cocchiarella F, Di Agostino S, Basile V, Gostissa M (2005). Direct p53 transcriptional repression: in vivo analysis of CCAAT-containing G2/M promoters. Mol Cell Biol.

[24] Werner H, Karnieli E, Rauscher FJ, LeRoith D (1996). Wild-type and mutant p53 differentially regulate transcription of the insulin-like growth factor I receptor gene. Proc Natl Acad Sci USA.

[25] Tschaharganeh DF, Xue W, Calvisi DF, Evert M, Michurina TV, Dow LE (2014). p53-dependent nestin regulation links tumor suppression to cellular plasticity in liver cancer. Cell.

[26] Mitchell S, Vargas J, Hoffmann A (2016). Signaling via the NFκB system. Wiley Interdiscip Rev Syst Biol Med.

[27] Napetschnig J, Wu H (2013). Molecular basis of NF-κB signaling. Annu Rev Biophys.

[28] Hinz M, Scheidereit C (2014). The IκB kinase complex in NF-κB regulation and beyond. EMBO Rep.

[29] Chariot A (2009). The NF-κB-independent functions of IKK subunits in immunity and cancer. Trends Cell Biol.

[30] Huang W, Huang M (2013). Beyond NF-κB activation: nuclear functions of IκB kinase α. J. Biol Med Sci.

[31] Dong W, Li Y, Gao M, Hu MR, Li XG, Mai SY (2012). IKKα contributes to UVB-induced VEGF expression by regulating AP-1 transactivation. Nucleic Acids Res.

[32] Li Y, Hao Y, Gao M, Dong W, Hu MR, Yuan ST (2011). IKKβ downregulation is critical for triggering JNKs-dependent cell apoptotic response in the human hepatoma cells under arsenite exposure. Mol Cell Biochem.

[33] Song L, Dong W, Gao M, Li JX, Hu MR, Guo N (2010). A novel role of IKKα in the mediation of UVB-induced G0/G1 cell cycle arrest response by suppressing Cyclin D1 expression. Biochim Biophys Acta.

[34] Feng JH, Tamaskovic R, Yang ZZ, Brazil DP, Merlo A, Hess D (2004). Stabilization of Mdm2 via Decreased Ubiquitination Is Mediated by Protein Kinase B/Akt-dependent Phosphorylation. J Biol Chem.

[35] Song L, Li J, Hu MR, Huang CS (2008). Both IKKα and IKKβ are implicated in the arsenite-induced AP-1 transactivation correlating with cell apoptosis through NF-κB activity-independent manner. Exp Cell Res.

[36] Tergaonkar V, Pando M, Vafa O, Wahl G, Verma I (2002). p53 stabilization is decreased upon NFκB activation: a role for NFκB in acquisition of resistance to chemotherapy. Cancer Cell.

[37] Xu XD, Wang HL, Liu SS, Xing C, Liu Y, Aodengqimuge (2016). TP53-dependent autophagy links the ATR-CHEK1 axis activation to proinflammatory VEGFA production in human bronchial epithelial cells exposed to fine particulate matter (PM2.5). Autophagy.

[38] Song L, Li JX, Ye JP, Yu G, Ding J, Zhang DY (2007). p85α acts as a novel signal transducer for mediation of cellular apoptotic response to UV radiation. Mol Cell Biol.

