# Supplementary material for: Transcriptional repression of IKKβ by p53 in arsenite-induced GADD45α accumulation and apoptosis
Source: Oncogene. 2018 Sep 3;38(5):731–46. doi: 10.1038/s41388-018-0478-7 (PMC6355650; doi:10.1038/s41388-018-0478-7)
Supplement: Supplementary file 1 — supplemented data [file 41388_2018_478_MOESM1_ESM.pdf]

Transcriptional repression of IKKβ by p53 in arsenite-induced GADD45α accumulation and apoptosis

Yongliang Hu[1,*], Rui Jin[4,*], Ming Gao[1,5,*], Huan Xu[1], Shuxian Zou[1,2], Xiaoguang Li[1], Chen Xing[1], Qiyu Wang[1], Hongli Wang[1], Jiannan Feng[1], Meiru Hu[1], Lun Song[#, 1, 2, 3]

Hu Y, et al. Figure S1

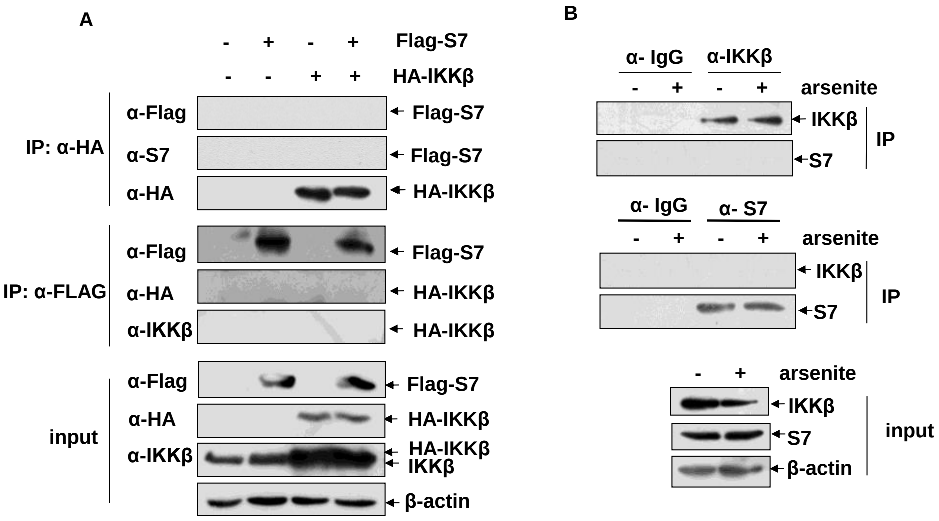

Figure S1: IKKβ neither interacts with S7 nor regulates its expression level under both resting and arsenite exposure conditions. (A) HepG2cells were either left untreated or transfected with the expression plasmid encoding FLAG-S7 or in combination with HA-IKKβ construct. Cell lysate were immunoprecipitated with anti-HA or anti-FLAG antibody and then the immunoprecipitants were probed with the antibodies as indicated. (B) HepG2 cells were left untreated or treated with arsenite (20 μM) for 6 h. Cell lysate were immunoprecipitated with anti-IKKβ, anti-S7 antibody or the control IgG, and then the immunoprecipitants were probed with the antibodies as indicated.

Hu Y, et al. Figure S2

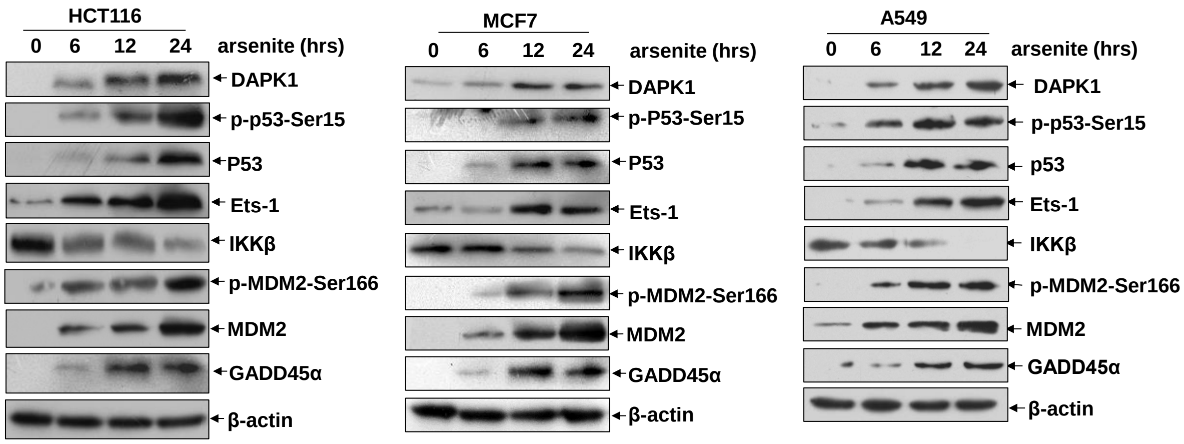

Figure S2: DAPK1/p53/Ets-1/IKKβ/MDM2/GADD45α pathway is activated by arsenite in different cancer cell lines. HCT116, MCF7 and A549 cells were left untreated or treated with arsenite (20 μM). Then the activation of the DAPK1/p53/Ets-1/IKKβ/MDM2/GADD45α pathway was detected at 12 h after arsenite exposure.

Hu Y, et al. Figure S3

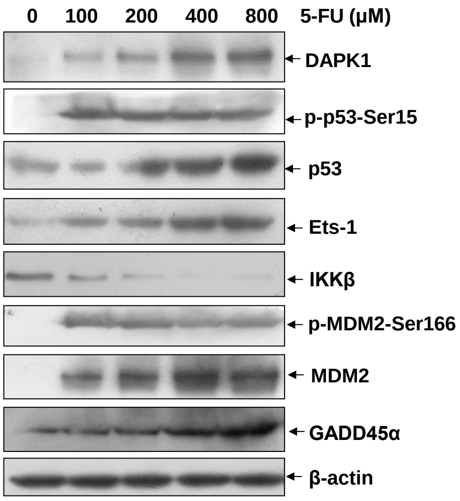

Figures S3: DAPK1/p53/Ets-1/IKKβ/MDM2/GADD45α pathway is activated by 5-Fu in HepG2 cells. HepG2 cells were left untreated or treated with different doses of 5-Fu as indicated. Then the activation of the DAPK1/p53/Ets-1/IKKβ/MDM2/GADD45α pathway was detected at 12 h after 5-Fu exposure.
